# Associations of Uric Acid with Polymorphisms in the δ-Aminolevulinic Acid Dehydratase, Vitamin D Receptor, and Nitric Oxide Synthase Genes in Korean Lead Workers

**DOI:** 10.1289/ehp.7927

**Published:** 2005-06-27

**Authors:** Virginia M. Weaver, Brian S. Schwartz, Bernard G. Jaar, Kyu-Dong Ahn, Andrew C. Todd, Sung-Soo Lee, Karl T. Kelsey, Ellen K. Silbergeld, Mark E. Lustberg, Patrick J. Parsons, Jiayu Wen, Byung-Kook Lee

**Affiliations:** 1 Division of Occupational and Environmental Health, Department of Environmental Health Sciences, Johns Hopkins University Bloomberg School of Public Health, Baltimore, Maryland, USA; 2 Department of Medicine, Johns Hopkins University School of Medicine, Baltimore, Maryland, USA; 3 Department of Epidemiology, Johns Hopkins University Bloomberg School of Public Health, Baltimore, Maryland, USA; 4 Institute of Industrial Medicine, SoonChunHyang University, Asan, South Korea; 5 Department of Community and Preventive Medicine, Mount Sinai School of Medicine, New York, New York, USA; 6 Department of Cancer Cell Biology, Harvard School of Public Health, Boston, Massachusetts, USA; 7 Division of Environmental Health Engineering, Department of Environmental Health Sciences, Johns Hopkins University Bloomberg School of Public Health, Baltimore, Maryland, USA; 8 Department of Epidemiology and Preventive Medicine, University of Maryland School of Medicine, Baltimore, Maryland, USA; 9 Lead Poisoning/Trace Elements Laboratory, Wadsworth Center, New York State Department of Health, Albany, New York, USA

**Keywords:** δ-aminolevulinic acid dehydratase, endothelial nitric oxide synthase, genetic susceptibility factors, kidney function, lead exposure, uric acid, vitamin D receptor

## Abstract

Recent research suggests that uric acid may be nephrotoxic at lower levels than previously recognized and that it may be one mechanism for lead-related nephrotoxicity. Therefore, in understanding mechanisms for lead-related nephrotoxicity, it would be of value to determine whether genetic polymorphisms that are associated with renal outcomes in lead workers and/or modify associations between lead dose and renal function are also associated with uric acid and/or modify associations between lead dose and uric acid. We analyzed data on three such genetic polymorphisms: δ-aminolevulinic acid dehydratase (*ALAD*), endothelial nitric oxide synthase (*eNOS*), and the vitamin D receptor (*VDR*). Mean (± SD) tibia, blood, and dimercaptosuccinic acid–chelatable lead levels were 37.2 ± 40.4 μg/g bone mineral, 32.0± 15.0 g/dL, and 0.77± 0.86 μg/mg creatinine, respectively, in 798 current and former lead workers. Participants with the *eNOS Asp* allele had lower mean serum uric acid compared with those with the *Glu/Glu* genotype. Among older workers (age ≥ median of 40.6 years), *ALAD* genotype modified associations between lead dose and uric acid levels. Higher lead dose was significantly associated with higher uric acid in workers with the *ALAD1-1* genotype; associations were in the opposite direction in participants with the variant *ALAD1-2* genotype. In contrast, higher tibia lead was associated with higher uric acid in those with the variant *VDR B* allele; however, modification was dependent on participants with the *bb* genotype and high tibia lead levels. We conclude that genetic polymorphisms may modify uric acid mediation of lead-related adverse renal effects.

Increasing evidence suggests that lead exposure may contribute to decreased renal function (Kim et al. 1996; Lin et al. 1999, 2003; Muntner et al. 2003; Payton et al. 1994; Staessen et al. 1992; Yu et al. 2004) and increased uric acid levels (Lin et al. 2002, 2001; Shadick et al. 2000) at lower doses than previously recognized, particularly in susceptible populations. Similarly, an increasing body of literature suggests that uric acid may be nephrotoxic at lower levels than currently appreciated (Johnson et al. 2003). In previous analyses in the same population of lead workers reported here, we found associations between lead dose and increased uric acid in older workers and evidence that uric acid may mediate some lead-related adverse renal effects (Weaver et al. 2005). Therefore, to understand the mechanisms of lead-related nephrotoxicity, it would be helpful to determine whether genetic polymorphisms that are associated with renal outcomes in lead workers and/or modify associations between lead dose and renal function are also associated with uric acid and/or modify associations between lead dose and uric acid. In this study we evaluated three such genetic polymorphisms: δ-aminolevulinic acid dehydratase (*ALAD*), the *Bsm*I polymorphism of the vitamin D receptor (*VDR*) gene, and the Glu298Asp polymorphism of the endothelial nitric oxide synthase (*eNOS*) gene. We examined associations of these polymorphisms and their effect modification on associations of three lead dose biomarkers in models of uric acid in a cross-sectional analysis of data from the first of three evaluations of 798 current and former Korean lead workers in a longitudinal study of the adverse health effects of inorganic lead exposure.

## Materials and Methods

### Study design and population.

We performed a cross-sectional analysis of first evaluation data from 798 current and former lead workers enrolled in a longitudinal study of the adverse health effects of inorganic lead exposure. All participants provided written, informed consent. The study protocol was approved by institutional review boards at the SoonChunHyang University and the Johns Hopkins University Bloomberg School of Public Health. Participation in the study was voluntary, and workers were paid approximately $30 for their time and effort. As previously described (Schwartz et al. 2001; Weaver et al. 2003a), workers were recruited from 26 different facilities including lead battery, lead oxide, lead crystal, and radiator manufactures and secondary lead smelting. No medical exclusionary criteria (e.g., blood pressure, renal disease) were applied. Study participants were not currently occupationally exposed to other known renal toxicants.

### Data collection.

Data collection was completed either at the Institute of Industrial Medicine of the SoonChunHyang University in Chonan or at the study plants, using previously reported methods (Schwartz et al. 2001; Weaver et al. 2003a). Data and biologic specimens collected included a standardized questionnaire on demographics and medical and occupational history; blood pressure; height and weight measurement; a blood specimen [for blood lead, blood urea nitrogen (BUN), serum creatinine, uric acid, and genotyping]; a spot urine sample [for *N*-acetyl-β-d-glucosaminidase (NAG), retinol-binding protein (RBP), and creatinine]; and tibia lead concentration. A 4-hr urine collection after oral administration of 10 mg/kg dimercaptosuccinic acid (DMSA) was also obtained to measure DMSA-chelatable lead and creatinine clearance (787 participants completed this collection).

### Laboratory methods.

We measured lead biomarkers and renal outcomes using previously reported methods (Schwartz et al. 2001; Weaver et al. 2003a). In brief, blood lead was measured (Fernandez 1975) with a Zeeman background-corrected atomic absorption spectrophotometer (model 8100; Hitachi Ltd. Instruments, Tokyo, Japan) at the Institute of Industrial Medicine, a certified reference laboratory for lead in Korea. Tibia lead was assessed via a 30-min measurement of the left midtibia diaphysis using ^109^Cd to fluoresce the K-shell X-rays of lead. The lead X-rays were recorded with a radiation detector and then quantified and compared with calibration data to estimate the concentration of lead in bone (Todd and Chettle 1994; Todd and McNeill 1993). All point estimates, including negative values, were retained in the statistical analyses, to minimize bias and to avoid censoring of data (Kim et al. 1995). Urine lead levels in the 4-hr collection were measured at the Wadsworth Center of the New York State Department of Health (Albany, NY, USA) by electrothermal atomic absorption spectrometry with Zeeman background correction (model 4100ZL; Perkin-Elmer, Norwalk, CT, USA) (Parsons and Slavin 1999).

We measured BUN, serum creatinine, and uric acid using an automatic chemical analyzer (model TBA 40FR biochemical analyzer; Toshiba, Tokyo, Japan). Urine creatinine was measured with the Sigma kit (Sigma Chemical Company, St. Louis, MO, USA). Measured creatinine clearance was defined as [(urinary creatinine in milligrams per deciliter × urine volume in milliliters) ÷ serum creatinine in milligrams per deciliter] ÷ collection time in minutes. Calculated creatinine clearance was obtained from the Cockcroft-Gault equation (Cockcroft and Gault 1976). We measured NAG using the PPR NAG test kit (PPR Diagnostics, Ltd., London, UK), and RBP was measured using a modification of the method of Topping et al. (1986).

We isolated DNA for genotyping from whole blood samples using the QIAamp blood kit (QIAGEN, Hilden, Germany). The *ALAD* polymorphism assayed (δ-aminolevulinic acid dehydratase NCBI accession no. AY319481) results in two alleles: *ALAD1* and the variant, *ALAD2*, which has a G-to-C transversion at codon 177. The protocol for *ALAD* genotyping involved an initial amplification that generated a 916 bp fragment (Schwartz et al. 1995; Wetmur et al. 1991; Ziemsen et al. 1986). A second amplification, using a pair of nested primers (kindly provided by J. Wetmur), generated an 887 bp fragment. The amplified fragment was cleaved at the diagnostic *Msp*1 site on the *ALAD2* allele. The Glu298Asp polymorphism of the *eNOS* gene (endothelial nitric oxidase synthase; NCBI accession no. X76307) involves a G-to-T transversion at nucleotide position 894 within exon 7, which results in substitution of aspartic acid for glutamic acid at codon 298; the variant allele is referred to as the *Asp* or *T* allele. Genotype was determined by a modification of the assay of Hibi et al. (1998) as previously described (Weaver et al. 2003b). The *VDR Bsm*I polymorphic site in intron 8 [vitamin D (1,25-dihydroxyvitamin D_3_) receptor; NCBI accession no. AY342401] consists of the common allele, denoted *b*, and a variant, denoted *B*, in which the *Bsm*I restriction enzyme site is absent. Amplification used primers originating in exon 7 and intron 8 as previously published (Schwartz et al. 2000b).

### Statistical analysis.

The primary goals of our analysis were to examine associations of *ALAD*, *VDR*, and *eNOS* genotype with uric acid in lead workers while controlling for other covariates and to evaluate whether *ALAD*, *VDR*, and *eNOS* genotypes modified associations between the lead dose biomarkers (tibia lead, blood lead, DMSA-chelatable lead) and uric acid while controlling for other covariates. Statistical analysis was completed using SAS software, version 8.2 (SAS Institute, Cary, NC, USA).

Initially, we examined variable distributions. The distributions of NAG and RBP showed departures from normality and were thus ln-transformed; the adequacy of this transformation was subsequently confirmed by examination of residuals from the final regression models. Linear regression modeling with a dichotomous genotype variable was used to compare uric acid by genotype, while controlling for the same covariates used in the interaction models. For *ALAD*, participants with the *ALAD1-2* genotype were compared with the reference group of participants with *ALAD1-1* genotype. Because of small numbers, all analyses combined homozygous and heterozygous variant genotype carriers for *VDR* (*BB* and *Bb*) and *eNOS* (*Glu/Asp* and *Asp/Asp*). Linear regression modeling with genotype and lead variable cross-product terms was used to evaluate effect modification by genotype on associations between lead biomarkers and uric acid. Models with tibia lead were repeated after removal of data from participants with the common allele whose tibia lead levels were higher than the range in those with the variant allele. Covariate selection for final regression models used a priori variables [age, sex, body mass index (BMI; defined as weight in kilograms divided by the square of height in meters)] in modeling that included other biologically relevant variables in separate models, as previously described (Weaver et al. 2005). Covariates retained included age, sex, BMI, categorical alcohol consumption (current, former, never), and, when noted in specific models, systolic blood pressure and serum creatinine. Models are presented with and without these last two variables because their interrelatedness with lead dose and uric acid suggests that neither modeling approach is entirely adequate (Weaver et al. 2005). Continuous independent variables were centered at the mean.

Because effect modification by age on associations between lead biomarkers and uric acid was observed in this population (Weaver et al. 2005), we dichotomized the population by median age and repeated both types of models described above (main effect and interaction) to evaluate associations of genotype in models of uric acid and effect modification by genotype on associations between lead biomarkers and uric acid in different age groups. Last, a model that included cross-products of tibia lead and combined *ALAD*/*VDR* genotype subsets was evaluated in participants in the older half of the population to determine if the genotype association previously reported in this population (lead workers with the *ALAD1-1* genotype were statistically less likely to have the *VDR bb* genotype; Schwartz et al. 2000a) was a factor in the effect modification observed in this study. Only two participants in the older age group had both variant alleles (*ALAD1-2*/*VDR Bb* or *BB*), which precluded meaningful analysis of this group; this subset was removed from the model. The final model assessed differences in the slopes of the relations between tibia lead and uric acid in the group of participants with the *ALAD1-2*/*VDR bb* genotypes and the *ALAD1-1*/*VDR Bb* or *BB* genotypes, in separate cross-product terms, compared with the reference group of participants with the *ALAD1-1* and *VDR bb* genotypes.

As in previous analyses (Weaver et al. 2003a), we evaluated models for linear regression assumptions and the presence of outlying points using added variable plots (Weisberg 1985), which are graphical summaries of the relation between *y* and a particular *x* adjusted for the other covariates. For each plot, two lines were overlaid: the regression line, and a line determined by a scatter plot smoothing method (lowess) that calculates a locally weighted least squares estimate for each point in the scatter plot (Cleveland 1979). This allows an examination of the data for outliers that are overly influential, as evidenced by inconsistency between the lowess and regression lines. When warranted, models were repeated without outliers. Models were also assessed for collinearity by examining variance inflation factors and conditional indices.

## Results

### Selected demographics, exposure, and health outcome measures.

A total of 79 (9.9%) participants were heterozygous for the *ALAD2* allele, and none was homozygous (Table 1). For *VDR*, 85 (10.7%) were genotype *Bb*, and 4 (0.5%) were *BB*. For *eNOS*, 114 (14.4%) participants were genotype *Glu/Asp*, and 6 (0.7%) were *Asp/Asp*. Mean (± SD) and frequencies for selected demographic, exposure, and outcome variables are presented by genotype in Table 1. Mean uric acid was normal in all genotype subsets. Medians for selected measures are presented by genotype in groups dichotomized by median age (Table 2).

#### ALAD.

**Figure 1:**
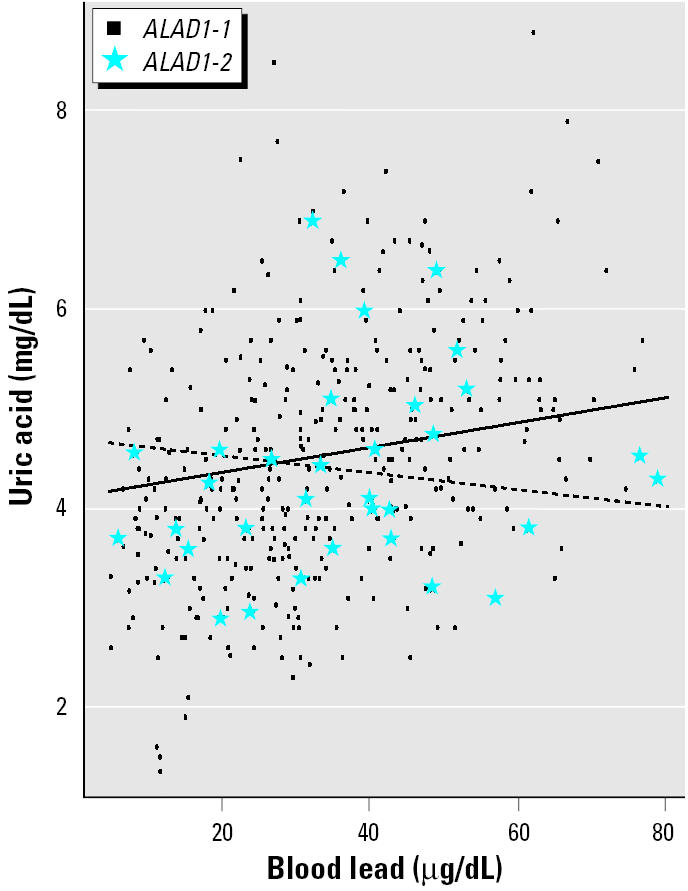
Plot of model assessing effect modification by *ALAD* genotype on the association of blood lead and uric acid in Korean lead workers whose ages are ≥ 40.6 years (Table 3, panel 2). Regression lines, generated using mean values of covariates in the model (age, sex, BMI, and alcohol use), are overlaid on crude data. The solid regression line represents the adjusted relation between blood lead and uric acid in older participants with the *ALAD1-1* genotype (circles); the dashed regression line represents the adjusted relation between blood lead and uric acid in older participants with the *ALAD1-2* genotype (stars).

**Figure 2:**
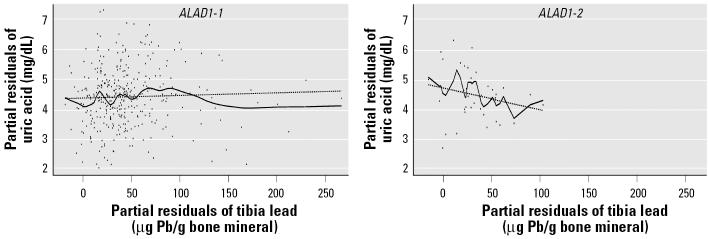
Added variable plot of the model assessing effect modification by *ALAD* genotype on the association between tibia lead and uric acid in Korean lead workers ≥ 40.6 years of age (Table 3, panel 2). For each plot, the regression line (dashed line) and the lowess line (solid line) of the partial residual data points, adjusted for age, sex, BMI, and alcohol use, are overlaid. For ease of interpretation, axes have been scaled so that the plotted residuals are centered around mean uric acid and tibia lead, rather than around zero.

Mean uric acid did not differ by *ALAD* genotype in the total population or in either age group (dichotomized by median age of 40.6 years). Among all participants, *ALAD* genotype modified the association between blood lead and uric acid. β-Coefficients were 0.0047 (*p* = 0.10) for blood lead in the reference group of those with the *ALAD1-1* genotype and −0.0158 (*p* = 0.05) for the cross-product term of *ALAD1-2* genotype and blood lead, respectively (thus, the slope of the relation between blood lead and uric acid in participants with the *ALAD1-2* genotype was −0.0111). This effect was confined to workers at or above the median age (Table 3, panels 1 and 2, blood lead models). The inverse nature of these associations by genotype, from the blood lead model in panel 2 of Table 3, is shown graphically in Figure 1. Borderline effect modification on the association between tibia lead and uric acid was also observed in older participants (Table 3, panel 2, tibia lead models). Consistent with known mechanisms for the hyperuricemic effects of lead (Weaver et al. 2005), β-coefficients decreased after additional adjustment for blood pressure and renal function (Table 3, panel 3). Results were similar when calculated creatinine clearance was added to these models instead of serum creatinine (data not shown). Added variable plots (Figure 2) of partial residuals of the tibia lead model (Table 3, panel 2) suggested different tibia lead level ranges by genotype. In order to compare differences over a similar range, data from 43 participants in the *ALAD1-1* group with tibia lead levels > 89 μg Pb/g bone mineral (levels above the crude range in participants with the *ALAD1-2* genotype) were removed. A positive association between tibia lead and uric acid in those with the *ALAD1-1* genotype (Table 3, truncated tibia lead models, panel 2) was then observed.

#### VDR.

**Figure 3:**
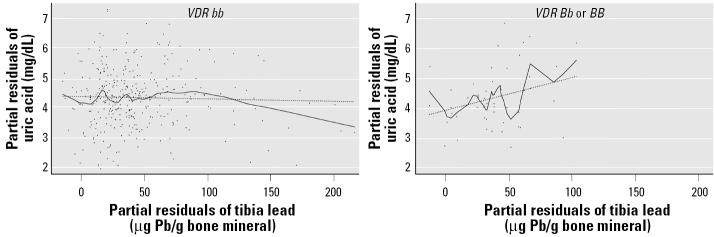
Added variable plot of the model assessing effect modification by *VDR* genotype on the association between tibia lead and uric acid in Korean lead workers ≥ 40.6 years of age (median) (Table 4, panel 2). For each plot, the regression line (dashed line) and the lowess line (solid line) of the partial residual data points, adjusted for age, sex, BMI, and alcohol use, are overlaid. For ease of interpretation, axes have been scaled so that the plotted residuals are centered around mean uric acid and tibia lead, rather than around zero.

Similar to *ALAD*, *VDR* genotype was not associated with uric acid levels, and effect modification was confined to older lead workers (Table 4, tibia lead models, panels 1 and 2). However, in contrast to *ALAD*, tibia lead was positively associated with uric acid in those participants with the variant *VDR B* allele, and effect modification on associations between blood lead and uric acid was not observed (Table 4). Modeling that included cross-products of tibia lead with combined genotype subsets compared with a reference group of participants with both the *ALAD1-1* and *VDR bb* genotypes revealed that the opposite direction associations between tibia lead and uric acid in participants with the *VDR B* allele compared with those with the *ALAD1-2* allele were not simply due to the genotype associations previously reported in this population (lead workers with the *ALAD1-1* genotype were statistically less likely to have the *VDR bb* genotype; Schwartz et al. 2000a) (Table 5). Similar to *ALAD*, the range of tibia lead levels differed by genotype (Figure 3). In contrast, when data from 27 participants with the *VDR bb* genotype with tibia lead levels > 103 μg Pb/g bone mineral (above the range in participants with *VDR Bb* or *BB* genotypes) were removed in order to compare differences over a similar range, effect modification was no longer significant (Table 4, truncated tibia lead models, panel 2).

#### eNOS.

In contrast to *ALAD* and *VDR*, the *eNOS* genotype was associated with uric acid levels. Mean serum uric acid was lower in participants with the *eNOS Asp* allele compared with those with the *Glu/Glu* genotype (β= −0.1913; SE β= 0.0932; *p* = 0.04; adjusted for age, sex, BMI, serum creatinine, systolic blood pressure, and blood lead). Additional modeling in two groups, dichotomized by median age, revealed that this association was confined to participants in the older half of the population. No effect modification by *eNOS* genotype on associations of lead biomarkers and uric acid was observed.

## Discussion

We evaluated whether polymorphisms in three genes (*ALAD*, *VDR*, and *eNOS*) were associated with uric acid or modified relations of lead biomarkers with uric acid in a cross-sectional analysis of data from the first evaluation in a longitudinal study of 798 current and former Korean lead workers. After adjustment, participants with the *eNOS Asp* allele had lower mean uric acid. Effect modification by *ALAD* on associations between lead dose and uric acid was observed in participants whose ages were in the older half of the age range. Higher lead dose was associated with higher uric acid in workers with the *ALAD1-1* genotype; associations were in the opposite direction in participants with the *ALAD1-2* genotype. *VDR* genotype modified the association of tibia lead and uric acid, also in the older half of the population. However, in contrast to *ALAD*, higher tibia lead was associated with higher uric acid in those with the variant *B* allele, but this effect was dependent on data from participants with the *bb* genotype and high tibia lead levels.

The ALAD enzyme is a principal lead-binding protein. The isozymes in those with the variant *ALAD2* allele bind a greater proportion of blood lead than does the isozyme in individuals with the *ALAD1-1* genotype (Bergdahl et al. 1997). In the population that is the focus of this report, mean blood lead was higher in participants with the *ALAD1-2* genotype compared with those with the *ALAD1-1* genotype (Schwartz et al. 2000a). However, neither tibia nor DMSA-chelatable lead levels differed significantly. Other studies have also reported that participants with the *ALAD2* allele have higher blood lead levels than do those with the *ALAD1-1* genotype, although studies in populations with mean blood lead levels < 10 μg/dL have generally not reported a difference (Hu et al. 2001; Kamel et al. 2003; Kelada et al. 2001). The limited data on associations of *ALAD* genotype with bone lead levels reveal no difference in some studies (similar to results in our population) (Fleming et al. 1998; Smith et al. 1995). Lower cortical (tibia) and/or trabecular (patella, calcaneus) bone lead levels in those with the *ALAD2* allele have been reported in others (Hu et al. 2001; Kamel et al. 2003). Other toxicokinetic differences have also been reported in participants with the *ALAD2* allele (Fleming et al. 1998; Hu et al. 2001; Schwartz et al. 1997; Smith et al. 1995). Overall, these data suggest that tighter binding of lead by the isozymes of the *ALAD2* allele decreases lead sequestration in bone. Therefore, the impact on toxicity would depend on whether enzyme bound lead is bioavailable and more toxic than lead that is stored in bone and subsequently released. As a result, toxicity may differ by target organ.

Two studies have examined the impact of *ALAD* genotype on serum uric acid levels in lead exposed populations. In a study of 691 construction workers, whose mean blood lead was 7.8 μg/dL, Smith et al. (1995) found borderline (*p* = 0.09) higher mean uric acid after adjustment for age, alcohol ingestion, and blood lead in the 96 participants with the *ALAD2* allele compared with those with the *ALAD1-1* genotype. Effect modification was not evaluated. Wu et al. (2003) found no difference in uric acid by genotype in 709 participants in the Normative Aging Study (mean blood lead = 6.2 μg/dL). However, effect modification by genotype on associations of patella and tibia lead with uric acid of borderline significance (*p* < 0.1) was observed; β-coefficients (and slopes) were greater in those with the variant allele. This difference was significant in participants whose patella lead levels were > 15 μg/g bone mineral (*p* = 0.04).

Synthesizing our data with these studies is complicated by the inverse associations we observed. Similar inverse relations between blood and DMSA-chelatable lead and renal outcomes in participants with the *ALAD2* allele were previously reported in this population (Weaver et al. 2003b). Because uric acid is filtered at the glomerulus, levels in serum are also influenced by renal function. Therefore, the associations between lead dose and uric acid may be due to the same process causing inverse associations between lead dose and renal function in those with the *ALAD2* allele. If this process represents lead-related hyperfiltration, the associations between lead dose and uric acid may become positive as this population ages and eventually, as in the Normative Aging Study, be stronger in those with the *ALAD2* allele than in those with the *ALAD1-1* genotype. However, several steps are involved in the renal handling of uric acid, including a secretion step in the proximal tubule, which is also thought to be adversely affected by lead. The fact that effect modification by *ALAD* genotype on associations between tibia lead and uric acid persists after adjustment for renal function suggests that one or more of the other renal handling processes for uric acid are involved. Analysis of our longitudinal data will be very helpful in understanding these complex relations.

The potential for uric acid–related nephrotoxicity must be considered in these associations, as well. When previously published models of effect modification by *ALAD* on associations between lead dose and renal function (Weaver et al. 2003b) were also controlled for uric acid, the effect modification observed remained statistically significant (data not shown), indicating that this effect is not mediated solely through uric acid. However, the current analyses do provide further evidence, in addition to our recently published results (Weaver et al. 2005), that lead dose increases uric acid in these workers, even after control for variables that are both confounders and mediators, such as blood pressure and renal function.

Polymorphisms of the gene encoding for the VDR are of interest in lead-exposed populations because of the importance of the receptor for calcium absorption and bone mineralization. These pathways affect lead absorption from the gastrointestinal tract and may affect lead storage and/or release from bone (Onalaja and Claudio 2000). In the lead workers studied in this report, participants with the *B* allele were found to have significantly higher blood, tibia, and DMSA-chelatable lead than did those with the *bb* genotype (Schwartz et al. 2000a). The effect modification of *VDR* genotype on the association of tibia lead and uric acid in participants who are in the older half of the population may reflect toxicodynamic differences. However, conclusions must be tempered by the fact that this effect is dependent on, at most, 27 participants with tibia lead levels > 103 μg Pb/g bone mineral who reduce the β estimate of the relation between tibia lead and uric acid in participants with the *bb* genotype. Further, no associations between *VDR* genotype and renal outcomes were observed, nor was consistent effect modification by *VDR* on associations between lead measures and renal outcomes present in this population (Weaver et al. 2003b). Interestingly, the shape of the lowess lines for the relations between tibia lead and uric acid in workers with the common genotypes (*ALAD1-1* and VDR *bb*; Figures 2 and 3) suggests differences at higher tibia lead levels that could reflect survivor bias. We plan to explore this further in longitudinal analyses.

eNOS catalyzes the transformation of l-arginine to the vasodilator, nitric oxide (NO). Animal models of kidney disease indicate that administration of l-arginine results in decreased renal damage that is thought to be mediated via increased NO (Klahr 2001). The functional significance of the Glu298Asp polymorphism remains uncertain. Some authors have reported decreased NO with the variant *Asp* allele (Noiri et al. 2002; Persu et al. 2002; Sofowora et al. 2001). However, others have reported no difference in various functional measures (Golser et al. 2003; Hoffmann et al. 2005; Li et al. 2004; Moon et al. 2002). If the *Asp* allele is ultimately shown to decrease NO, the variant may confer additional risk in lead exposure that also decreases NO (Vaziri 2002). In addition, this *eNOS* polymorphism may modify lead toxicokinetics. A recent study of data from the third evaluation of the lead workers in this report (*n* = 652) found that *eNOS* genotype modified the relation between patella lead and age; workers with the *Asp* allele had higher increases in patella lead with increasing age than did lead workers who were homozygous for the *Glu* allele (Theppeang et al. 2004). In the population that is the focus of this report, we found inconsistent associations of *eNOS* genotype with renal function (higher BUN and measured creatinine clearance in those with the *Asp* allele) (Weaver et al. 2003b). Effect modification was also noted; longer lead job duration was associated with higher serum creatinine and lower calculated creatinine clearance in participants with the *Asp* allele and with higher calculated creatinine clearance in participants with the *Glu/Glu* genotype (Weaver et al. 2003b). The lower uric acid observed in those with the *Asp* allele may indicate that, if this allele does confer increased renal risk in lead exposed populations, uric acid does not contribute further to that risk.

Our data suggest that all three genetic polymorphisms may affect uric acid in these lead workers. However, effect modification by *ALAD* genotype on associations between lead dose and uric acid was most consistent with the observed effect modification by this genotype on associations between lead dose and renal function. This work also suggests that effect modification by genotype is present only in the older half of the population. However, our ability to explore effect modification by genotype in different age groups is limited by the small number of participants with the variant alleles in these age-stratified models. As a result, even after removal of outliers, most data points in the variant groups are influential. Longitudinal data analysis will be useful, particularly in understanding the inverse associations seen with *ALAD*.

## Figures and Tables

**Table 1:** Selected demographic, exposure, and outcome variables by *ALAD*, *eNOS*, and *VDR* genotype in 798 Korean lead workers.^a^

	*ALAD*		*eNOS*		*VDR*	
Characteristic	1–1	1–2	*Glu/Glu*	*Asp/Glu* or *Asp/Asp*	*bb*	*Bb* or *BB*
No. (%)	716 (90.1)	79 (9.9)	673 (84.9)	120 (15.1)	709 (88.8)	89 (11.2)
Sex *n* (%)						
Male	569 (79.5)	62 (78.5)	537 (79.8)	93 (77.5)	572 (80.7)	62 (69.7)
Female	147 (20.5)	17 (21.5)	136 (20.2)	27 (22.5)	137 (19.3)	27 (30.3)
Alcohol use, *n* (%)						
No previous alcohol	207 (29.0)	24 (30.4)	201 (30.0)	30 (25.0)	208 (29.4)	23 (25.8)
Current use	466 (65.2)	49 (62.0)	429 (63.8)	84 (70.0)	456 (64.4)	62 (69.7)
Past use	42 (5.9)	6 (7.6)	42 (6.3)	6 (5.0)	44 (6.2)	4 (4.5)
BMI (kg/m^2^)	23.1 ± 3.0	22.3 ± 2.6	23.1 ± 3.0	22.7 ± 2.8	22.9 ± 2.9	23.9 ± 3.4
Age (years)	40.5 ± 10.2	40.1 ± 9.7	40.3 ± 10.1	41.1 ± 10.1	40.2 ± 10.1	42.7 ± 10.3
Systolic blood pressure (mm Hg)	123.4 ± 16.5	122.3 ± 14.5	123.4 ± 16.5	122.7 ± 15.5	122.6 ± 15.6	129.1 ± 20.6
Blood lead (μg/dL)	31.7 ± 14.9	34.2 ± 15.9	32.0 ± 15.1	31.2 ± 14.6	31.6 ± 14.8	34.8 ± 16.1
Tibia lead (μg Pb/g bone mineral)	37.5 ± 40.6	31.4 ± 29.5	37.5 ± 41.6	35.8 ± 34.0	37.1 ± 41.2	38.1 ± 33.5
DMSA-chelatable lead (μg Pb/mg creatinine)	0.76 ± 0.82	0.84 ± 1.19	0.78 ± 0.90	0.72 ± 0.62	0.75 ± 0.87	0.93 ± 0.76
Uric acid (mg/dL)	4.8 ± 1.2	4.6 ± 1.1	4.9 ± 1.2	4.6 ± 1.1	4.8 ± 1.2	4.7 ± 1.1
Serum creatinine (mg/dL)	0.90 ± 0.15	0.86 ± 0.12	0.90 ± 0.16	0.89 ± 0.13	0.90 ± 0.16	0.89 ± 0.13

*ALAD*, *eNOS*, and *VDR* genotyping were completed on 795, 793, and 798 lead workers, respectively. Modified from Weaver et al. (2003b).

a Values are mean ± SD unless otherwise noted.

**Table 2:** Medians of selected demographic, exposure, and outcome variables in Korean lead workers by *ALAD* and *VDR* genotype in two groups dichotomized by median age (40.6 years).

	*ALAD*				*VDR*			
	Age < 40.6 years		Age ≥ 40.6 years		Age < 40.6 years		Age ≥ 40.6 years	
Characteristic	*1–1*	*1–2*	*1–1*	*1–2*	*bb*	*Bb* or *BB*	*bb*	*Bb* or *BB*
No.^a^	355	42	361	37	360	38	349	51
Age (years)	32.8	33.3	48.6	49.4	32.7	33.7	48.3	49.7
Lead job duration (years)	3.9	4.1	9.7	9.5	3.8	4.2	9.8	8.3
Blood lead (μg/dL)	28.8	31.9	30.4	34.3	29.5	29.4	30.4	35.5
Tibia lead (μg Pb/g bone mineral)	20.9	22.1	30.7	25.6	20.9	25.4	29.4	35.1
DMSA-chelatable lead (μg Pb/mg creatinine)	0.39	0.54	0.64	0.64	0.39	0.60	0.62	0.82
Uric acid (mg/dL)	5.1	4.9	4.4	4.1	5.1	5.2	4.4	4.2
BUN (mg/dL)	13.9	12.4	14.4	13.7	13.7	13.1	14.4	14.0
Serum creatinine (mg/dL)	0.93	0.89	0.88	0.81	0.92	0.91	0.87	0.86
Measured creatinine clearance (mL/min)	121.8	119.0	101.4	108.9	121.5	118.1	103.3	100.6
Calculated creatinine clearance (mL/min)	102.9	102.0	83.4	85.4	102.8	101.6	83.6	83.1

a Actual value, not median.

**Table 3:** Linear regression models of uric acid, evaluating effect modification by *ALAD* genotype on associations of blood and tibia lead in two groups of lead workers, dichotomized by median age (40.6 years).

	Panel 1: age < 40.6 years			Panel 2: age ≥ 40.6 years			Panel 3: age ≥ 40.6 years		
Variable	β-Coefficient	SE β	*p*-Value	β-Coefficient	SE β	*p*-Value	β-Coefficient	SE β	*p*-Value
Blood lead models									
Intercept	4.4070	0.1776	< 0.01	4.7507	0.1108	< 0.01	4.5906	0.1078	< 0.01
Age (years)	−0.0348	0.0084	< 0.01	−0.0123	0.0098	0.21	−0.0188	0.0098	0.06
Systolic blood pressure (mm Hg)	—	—	—	—	—	—	0.0046	0.0027	0.09
Serum creatinine (mg/dL)	—	—	—	—	—	—	2.4921	0.3799	< 0.01
ALAD1-2	−0.2619	0.1591	0.10	−0.0292	0.1776	0.87	0.0870	0.1693	0.61
Blood lead (μg/dL)^a^	−0.0043	0.0040	0.27	0.0127	0.0040	< 0.01	0.0089	0.0038	0.02
Blood lead × *ALAD1-2*^b^	−0.0161	0.0134	0.23	−0.0212	0.0102	0.04	−0.0143	0.0098	0.14
Tibia lead models									
Intercept	4.3928	0.1746	< 0.01	4.7280	0.1075	< 0.01	4.6027	0.1074	< 0.01
Age (years)	−0.0335	0.0083	< 0.01	−0.0062	0.0096	0.52	−0.0157	0.0098	0.11
Systolic blood pressure (mm Hg)	—	—	—	—	—	—	0.0059	0.0027	0.03
Serum creatinine (mg/dL)	—	—	—	—	—	—	2.0767	0.4380	< 0.01
ALAD1-2	−0.3553	0.2330	0.13	−0.1993	0.2013	0.32	−0.1180	0.1955	0.55
Tibia lead (μg Pb/g bone mineral)^a^	−0.0044	0.0020	0.03	0.0009	0.0013	0.48	0.0002	0.0013	0.89
Tibia lead × *ALAD1-2*^b^	−0.0047	0.0103	0.65	−0.0151	0.0079	0.06	−0.0138	0.0077	0.07
Truncated tibia lead models^c^									
Intercept				4.8218	0.1159	< 0.01	4.6499	0.1159	< 0.01
Age (years)				−0.0074	0.0103	0.48	−0.0160	0.0103	0.12
Systolic blood pressure (mm Hg)				—	—	—	0.0058	0.0028	0.04
Serum creatinine (mg/dL)				—	—	—	2.3661	0.4620	< 0.01
ALAD1-2				−0.2763	0.2110	0.19	−0.1737	0.2033	0.39
Tibia lead (μg Pb/g bone mineral)^a^				0.0079	0.0027	< 0.01	0.0063	0.0026	0.02
Tibia lead × *ALAD1-2*^b^				−0.0214	0.0084	0.01	−0.0193	0.0080	0.02

—, model shown was not adjusted for that covariate. Panels 1 and 2 display results in the younger and older groups, respectively. Panel 3 shows results in the older group after additional control for systolic blood pressure and serum creatinine. Models were also adjusted for sex, BMI, and alcohol use.

a Reference category of homozygotes for the common gene allele *(ALAD1-1*).

b *p*-Values for the cross-product terms reflect the statistical significance of the difference between the slopes of the regression line for the variant gene group and the regression line for the reference gene group; slopes in the variant gene group are obtained by adding the β-coefficient of the cross-product term to the β-coefficient for the reference category [i.e., the slope of the relation between blood lead and uric acid in those with *ALAD1-2* genotype is −0.0085 in panel 2 (0.0127 + −0.0212)].

c Tibia lead levels > 89 μg Pb/g bone mineral were removed from models.

**Table 4:** Linear regression models of uric acid, evaluating effect modification by *VDR* genotype on associations of blood and tibia lead in two groups of lead workers, dichotomized by median age (40.6 years).

	Panel 1: age < 40.6 years			Panel 2: age ≥ 40.6 years			Panel 3: age ≥ 40.6 years		
	β-Coefficient	SE β	*p*-Value	β-Coefficient	SE β	*p*-Value	β-Coefficient	SE β	*p*-Value
Blood lead models									
Intercept	4.3906	0.1754	< 0.01	4.7529	0.1104	< 0.01	4.6087	0.1074	< 0.01
Age (years)	−0.0341	0.0083	< 0.01	−0.0118	0.0099	0.23	−0.0190	0.0099	0.06
Systolic blood pressure (mm Hg)	—	—	—	—	—	—	0.0056	0.0027	0.04
Serum creatinine (mg/dL)	—	—	—	—	—	—	2.1009	0.3302	< 0.01
*VDR Bb* or *BB*	0.0073	0.1654	0.97	−0.0447	0.1565	0.78	−0.1201	0.1502	0.42
Blood lead (μg/dL)^a^	−0.0056	0.0040	0.17	0.0111	0.0040	< 0.01	0.0087	0.0038	0.02
Blood lead × *VDR Bb* or *BB*^b^	0.0018	0.0109	0.87	−0.0041	0.0093	0.66	−0.0026	0.0089	0.77
Tibia lead models									
Intercept	4.3660	0.1756	< 0.01	4.7474	0.1070	< 0.01	4.6267	0.1064	< 0.01
Age (years)	−0.0338	0.0083	< 0.01	−0.0027	0.0097	0.78	−0.0122	0.0098	0.22
Systolic blood pressure (mm Hg)	—	—	—	—	—	—	0.0061	0.0027	0.02
Serum creatinine (mg/dL)	—	—	—	—	—	—	2.1603	0.4382	< 0.01
*VDR Bb* or *BB*	−0.0599	0.2093	0.78	0.0842	0.1515	0.58	−0.0140	0.1484	0.93
Tibia lead (μg Pb/g bone mineral)^a^	−0.0038	0.0021	0.07	−0.0008	0.0014	0.57	0.0015	0.0014	0.27
Tibia lead × *VDR Bb* or *BB*^b^	−0.0033	0.0083	0.69	0.0138	0.0062	0.03	0.0142	0.0060	0.02
Truncated tibia lead models^c^									
Intercept				4.7765	0.1110	< 0.01	4.6361	0.1108	< 0.01
Age (years)				−0.0057	0.0101	0.57	−0.0142	0.0101	0.16
Systolic blood pressure (mm Hg)				—	—	—	0.0061	0.0028	0.03
Serum creatinine (mg/dL)				—	—	—	2.2604	0.4534	< 0.01
*VDR Bb* or *BB*				0.0502	0.1555	0.75	−0.0608	0.1522	0.69
Tibia lead (μg Pb/g bone mineral)^a^				0.0036	0.0023	0.12	0.0026	0.0022	0.26
Tibia lead × *VDR Bb* or *BB*^b^				0.0100	0.0064	0.12	0.0106	0.0062	0.09

—, model shown was not adjusted for that covariate. Panels 1 and 2 display results in the younger and older groups, respectively. Panel 3 shows results in the older group after additional control for systolic blood pressure and serum creatinine. Models were also adjusted for sex, BMI, and alcohol use.

a Reference category of homozygotes for the common gene allele (*VDR bb*).

b *p*-Values for the cross-product terms reflect the statistical significance of the difference between the slopes of the regression line for the variant gene group and the regression line for the reference gene group; slopes in the variant gene group are obtained by adding the β-coefficient of the cross-product term to the β-coefficient for the reference category [i.e., the slope of the relation between tibia lead and uric acid in those with *VDR Bb* or *BB* genotypes is 0.0130 in panel 2 (−0.0008 + 0.0138)].

c Tibia lead levels > 103 μg Pb/g bone mineral were removed from models.

**Table 5:** Linear regression model evaluating effect modification by combined *ALAD* and *VDR* genotypes on the association between tibia lead and uric acid, in lead workers ≥ 40.6 years of age (median).

Blood lead models	β-Coefficient	SE β	*p*-Value
Intercept	4.6078	0.1086	< 0.01
Age (years)	−0.0133	0.0099	0.18
Systolic blood pressure (mm Hg)	0.0058	0.0027	0.03
Serum creatinine (mg/dL)	2.1352	0.4420	< 0.01
*ALAD1-2* and *VDR bb*	−0.1086	0.2067	0.60
*ALAD1-1* and *VDR Bb* or *BB*	0.0111	0.1533	0.94
Tibia lead (μg Pb/g bone mineral)^a^	−0.0008	0.0014	0.59
Tibia lead × *ALAD1-2* and *VDR bb*^b^	−0.0122	0.0079	0.12
Tibia lead × *ALAD1-1* and *VDR Bb* or *BB*^b^	0.0140	0.0061	0.02

Models were also adjusted for sex, BMI, and alcohol use.

a Reference category for homozygotes for both common gene alleles (*ALAD1-1* and *VDR bb*).

b *p*-Values for the cross-product terms reflect the statistical significance of the difference between the slopes of the regression line for the variant gene groups and the regression line for the reference gene group. Slopes in the variant gene groups are obtained by adding the

β-coefficient of the cross-product term to the β-coefficient for the reference category [i.e., the slope of the relation between tibia lead and uric acid in those with both the *ALAD1-1* and *VDR Bb* or *BB* genotypes is 0.0132 (−0.0008 + 0.0140)].
